# High-Plex and High-Throughput Digital Spatial Profiling of Non-Small-Cell Lung Cancer (NSCLC)

**DOI:** 10.3390/cancers12123551

**Published:** 2020-11-27

**Authors:** James Monkman, Touraj Taheri, Majid Ebrahimi Warkiani, Connor O’Leary, Rahul Ladwa, Derek Richard, Ken O’Byrne, Arutha Kulasinghe

**Affiliations:** 1 School of Biomedical Sciences, Faculty of Health and Institute of Health and Biomedical Innovation, Queensland University of Technology, Brisbane, QLD 4000, Australia; james.monkman@qut.edu.au (J.M.); connor.oleary@health.qld.gov.au (C.O.); derek.richard@qut.edu.au (D.R.); k.obyrne@qut.edu.au (K.O.); 2 Translational Research Institute, Woolloongabba, QLD 4102, Australia; 3 Cancer and Ageing Research Program, Translational Research Institute, Brisbane, QLD 4000, Australia; 4 Queensland Pathology, Herston, QLD 4006, Australia; Touraj.Taheri@health.qld.gov.au; 5 School of Medicine, University of Queensland, Brisbane, QLD 4102, Australia; rahul.ladwa@health.qld.gov.au; 6 School of Biomedical Engineering, University of Technology Sydney, Sydney, NSW 2007, Australia; majid.warkiani@uts.edu.au; 7 Princess Alexandra Hospital, Woolloongabba, QLD 4102, Australia; 8 Institute of Molecular Bioscience, University of Queensland, Brisbane, QLD 4072, Australia

**Keywords:** nanostring GeoMx digital spatial profiling, NSCLC, tumour microenvironment, spatial, tissue microarray, differential protein expression

## Abstract

**Simple Summary:**

Characterizing the tumour microenvironment (TME) has become increasingly important to understand the cellular interactions that may be at play for effective therapies. In this study, we used a novel spatial profiling tool, the Nanostring GeoMX Digital Spatial Profiler (DSP) technology, to profile non-small-cell lung cancer (NSCLC) for protein markers across immune cell typing, immune activation, drug targets, and tumour modules. Comparative analysis was performed between the tumour, adjacent tissue, and microenvironment to identify markers enriched in these areas with spatial resolution. Our study reveals that this methodology can be a powerful tool for determining the expression of a large number of protein markers from a single tissue slide.

**Abstract:**

Profiling the tumour microenvironment (TME) has been informative in understanding the underlying tumour–immune interactions. Multiplex immunohistochemistry (mIHC) coupled with molecular barcoding technologies have revealed greater insights into the TME. In this study, we utilised the Nanostring GeoMX Digital Spatial Profiler (DSP) platform to profile a non-small-cell lung cancer (NSCLC) tissue microarray for protein markers across immune cell profiling, immuno-oncology (IO) drug targets, immune activation status, immune cell typing, and pan-tumour protein modules. Regions of interest (ROIs) were selected that described tumour, TME, and normal adjacent tissue (NAT) compartments. Our data revealed that paired analysis (*n* = 18) of matched patient compartments indicate that the TME was significantly enriched in CD27, CD3, CD4, CD44, CD45, CD45RO, CD68, CD163, and VISTA relative to the tumour. Unmatched analysis indicated that the NAT (*n* = 19) was significantly enriched in CD34, fibronectin, IDO1, LAG3, ARG1, and PTEN when compared to the TME (*n* = 32). Univariate Cox proportional hazards indicated that the presence of cells expressing CD3 (hazard ratio (HR): 0.5, *p* = 0.018), CD34 (HR: 0.53, *p* = 0.004), and ICOS (HR: 0.6, *p* = 0.047) in tumour compartments were significantly associated with improved overall survival (OS). We implemented both high-plex and high-throughput methodologies to the discovery of protein biomarkers and molecular phenotypes within biopsy samples, and demonstrate the power of such tools for a new generation of pathology research.

## 1. Introduction

Non-small-cell lung cancer (NSCLC) accounts for 85% of lung cancers, and is the leading cause of cancer-related deaths [1]. Patients are often diagnosed at an advanced stage, where the immediate prognosis is poor, resulting in a five-year survival rate of less than 20% [2,3]. With the emerging success of immune checkpoint blockade leading to durable responses and prolonged survival in 15–40% of cases, there is now a need for predictive biomarkers to guide patient selection for targeted therapies [4]. The use of comprehensive tumoural information to inform clinical decision-making is becoming increasingly important [5,6,7,8,9]. Studies in the tumour microenvironment (TME) have revealed that a high degree of T-cell infiltration into the tumour provides fertile grounds for effective immunotherapies [10]. As such, the immune contexture (type, density, and location, as well as phenotypic and functional profile of immune cells) has been used to understand a greater depth of the tumour-immune cell interactions, which may provide cues into predictive biomarkers of the response to immune checkpoint therapy (anti PD-1/PD-L1) [11,12].

While traditional immunohistochemistry (IHC) techniques allow for the spatial profiling of cells in the tumour, this is often lost when tumours are analysed using bulk tissue genomic approaches. Moreover, the actual cellular proportions, cellular heterogeneity, and deeper spatial distribution are lacking in characterisation. Spatial and immunological composition with cellular status can aid in identifying micro-niches within the TME [13]. The classification of the immune context within the TME lays the foundation for addressing how the immunological composition and status (activated/suppressed) may dictate response to therapy. Therefore, to address this need, imaging and tissue sampling is required simultaneously to analyse tumour tissue and immune proteins with spatial resolution.

In this study, we used the Nanostring GeoMX Digital Spatial Profiler (DSP) to measure compartment-specific expression of proteins across immune cell profiling, immuno-oncology (IO), drug targets, immune activation status, immune cell typing, and pan-tumour protein modules. We found that in paired analysis of matched compartments, the TME was enriched for CD27, CD3, CD4, CD44, CD45, CD45RO, CD68, CD163, and VISTA relative to the tumour regions. Unmatched analysis indicated that the normal adjacent tissue (NAT) (*n* = 19) was significantly enriched in CD34, fibronectin, IDO1, LAG3, ARG1, and PTEN when compared to TME. Univariate Cox proportional hazard analysis indicated that the presence of cells expressing CD3 (hazard ratio (HR): 0.5, *p* = 0.018), CD34 (HR: 0.53, *p* = 0.004), and ICOS (HR: 0.6, *p* = 0.047) in tumour compartments was associated with improved overall survival (OS).

## 2. Methods

### 2.1. Tissue Microarray

This study has QUT Human Research Ethics Committee (UHREC) approval (#2000000494). The NSCLC Tissue Micro Array (TMA) (HLugA180Su03), containing 92 cases with concordant histologically normal adjacent tissue, was obtained from US Biomax, Inc. (Rockville, MD, USA), including associated clinical information. H&E images were demarcated by a pathologist for tumour and non-tumour regions in each core. The tissue microarray was purchased from US Biomax (commercial source). These companies keep the informed consent of the patient samples used to create the microarrays.

### 2.2. Nanostring GeoMX Digital Spatial Profiler: Tissue Microarray

The slides were profiled using Technology Access Program (TAP) by Nanostring Technologies (Seattle, WA, United States). In brief, immunofluorescent staining was performed on the TMA with tissue morphology markers (PanCK, CD3, CD45, and DAPI) in parallel with DNA-barcoded antibodies within the immune cell profiling, IO drug targets, immune activation status, immune cell typing, and pan-tumour protein panels, as shown in Table 1. Geometric (circular) and custom regions of interest (ROIs) were selected based on visualisation markers to generate tumour (PanCK+) and TME (PanCK−) areas, from which barcodes were liberated by UV light using the GeoMx DSP instrument, then hybridised and counted on the Ncounter system.

### 2.3. Nanostring GeoMX Digital Spatial Profiler: Data Analysis

Patient data presented in Table 2 was generated in R studio [14] using the package “gtsummary” [15]. Remote access to the GeoMx DSP analysis suite (GEOMX-0069) allowed inspection, quality control (QC), normalisation, and differential expression to be performed. Briefly, each ROI was tagged with metadata for its compartment and patient pairing, in order to allow pairwise comparisons. Raw data was exported and plotted in R using “ggplot2” [16] for raw counts, a signal relative to IgG controls, and an evaluation of the Pearson correlation coefficient (*R*) between normalisation parameters using the “ggpubr” package [17]. Normalisation using Histone H3 and S6 proteins was performed in GeoMx DSP analysis suite. Differential expression between paired compartments was evaluated by paired *t*-tests with a Benjamini–Hochberg correction, while differential expression between unpaired compartments was performed by a Mann–Whitney test with Benjamini–Hochberg correction, and results were plotted in R studio using “ggplot2”. Relative expression data was exported from the GeoMx DSP analysis suite, hierarchical clustering performed using the R package “complexHeatmap” [18], and univariate Cox proportional hazards regression was performed using “survivalAnalysis” [19] package.

## 3. Results

### 3.1. Region of Interest (ROI) Selection

**Figure 1:**
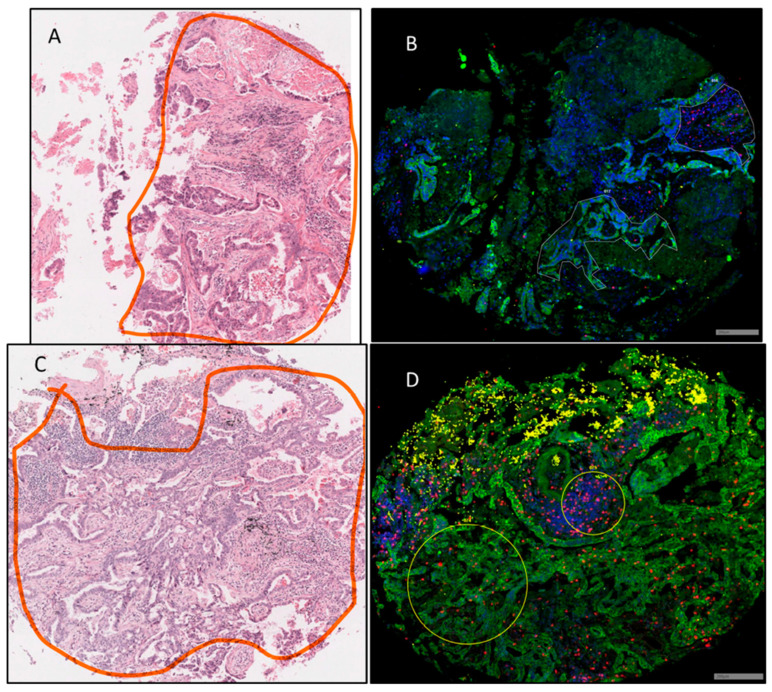
Representative H&E and immunofluorescent images of TMA cores. (**A**,**C**) Tumour regions of the cores were demarcated by a pathologist. (**B**,**D**) Corresponding regions of interest (ROIs) were captured for DSP analysis based on immunofluorescent staining for PanCK (Green), CD3 (Red), CD45 (Yellow), and DAPI (Blue). Tumour ROI (lower left) and tumour microenvironment (TME) ROI (upper right) were manually drawn in (**B**), and a circular tumour ROI (lower left) and TME ROI (upper right) are used in (**D**). Scale bar not available for H&E images, as images obtained from commercial supplier and are representative only. Scale bar = 200 µm.

**Figure 2:**
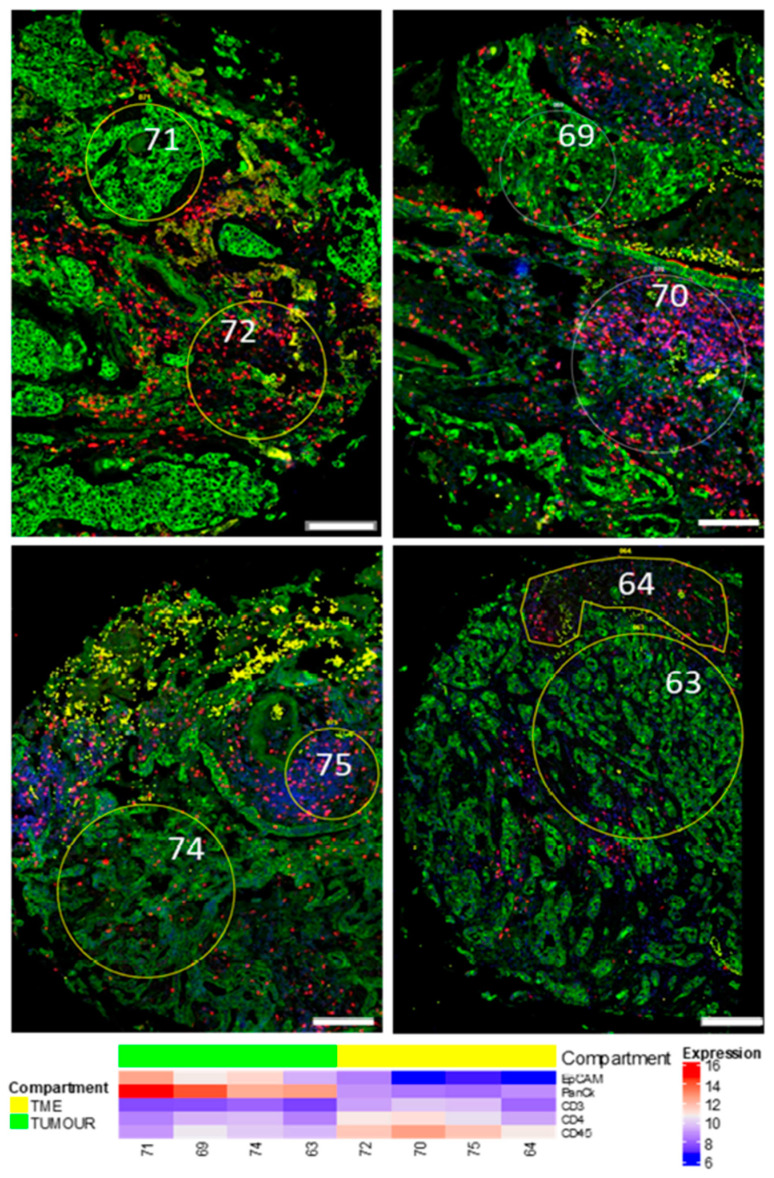
Tumour and immune ROIs exhibit distinct tumour/leukocyte marker expression. PanCK (Green), CD3 (Red), CD45 (Yellow), and DAPI (Blue). Representative paired tumour and immune ROIs are shown with corresponding Log2 expression of EpCAM, PanCK, CD3, CD4, and CD45. ROIs 71, 69, 74, and 63 represent tumour regions, while 72, 69, 74, and 63 are respective immune regions from corresponding patients. Scale bar = 200 µm.

Ninety-six ROIs in total were selected that were representative of 45 tumours, 32 TMEs, and 19 histologically normal adjacent tissues from the cohort of patients described in Table 2. Images of H&E-stained cores were demarcated by a pathologist and were utilised alongside Nanostring immunofluorescent staining for morphology markers PanCK, CD45, CD3, and DAPI to draw ROIs indicative of a tumour (CK+) or TME (CK-/CD3+). Figure 1 provides an example of this strategy where tumour and TME ROIs were able to be identified within the same tumour core. Of all the samples collected, comparisons from the same patient could be made between eight TME–NAT pairs, 14 NAT–tumour pairs, and 18 tumour–TME pairs. Figure 2 provides an overview of the tumour and immune ROI selection, as well as representative expression profiles for a number of associated markers.

### 3.2. Data Quality Control

**Figure 3:**
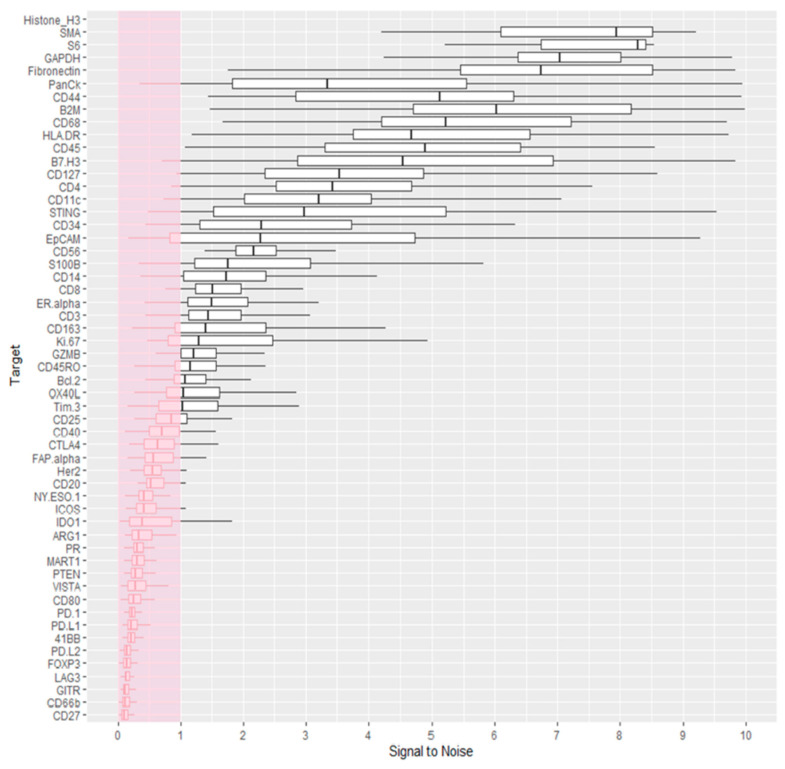
Probe counts relative to rabbit (Rb) and mouse (Ms) IgG controls. Counts of each probe were normalised to mean counts of Rb and Ms IgG within each ROI. The mean of these normalised values per probe was plotted to evaluate the robustness of the target protein signal to isotype background controls.

Quality control was performed within the GeoMx DSP analysis suite, to ensure the Ncounter quantification of probes was within specification. Raw probe counts per ROI were inspected to ensure comparable ranges of the signal, and to evaluate systemic variability ins sample groups. ROIs generated median counts within the range of 10^2^ and 10^3^, with observably lower median counts for ROIs 13, 67, and 96 (Figure S1). Raw probe counts were then inspected within TME, tumours, and NAT, as targets were expected to vary by respective tissue compartment (e.g., immune markers in TME vs. tumours). Robust counts were observed for abundant targets, including histone H3, SMA, S6, GAPDH, fibronectin, cytokeratin, CD44, CD68, β-2-microglobulin (B2M), HLA-DR, CD45, and B7-H3 (beyond axis range in Figure S2); however, the remaining probes shown exhibited raw counts below 200. Overall, raw counts from NAT ROIs shown in Figure S2 appeared to be lower than others for compartments, while tumour ROIs generated higher signals for most lowly-abundant probes. Of note, background isotype control IgG probes possessed counts between 50 to 150 (Figure S2), and while rabbit (Rb) IgG exhibited similar counts between tumour and TME compartments, mouse (Ms) IgG showed higher counts for tumours then TME. This suggests that background correction may not be the best strategy for the normalisation of lowly-expressed targets, as these targets are expressed at background or just above background levels, making their quantification challenging. Target signals relative to Ms and Rb IgG control probes was therefore evaluated to identify probes from which data should be considered with caution. Probes shown in Figure 3 whose median signal from all compartments relative to IgG was less than 1 (pink region) were thus followed with caution, and 31 of the 55 probes above CD25 in Figure 3 below were considered robust for further analysis.

### 3.3. Data Normalisation

**Figure 4:**
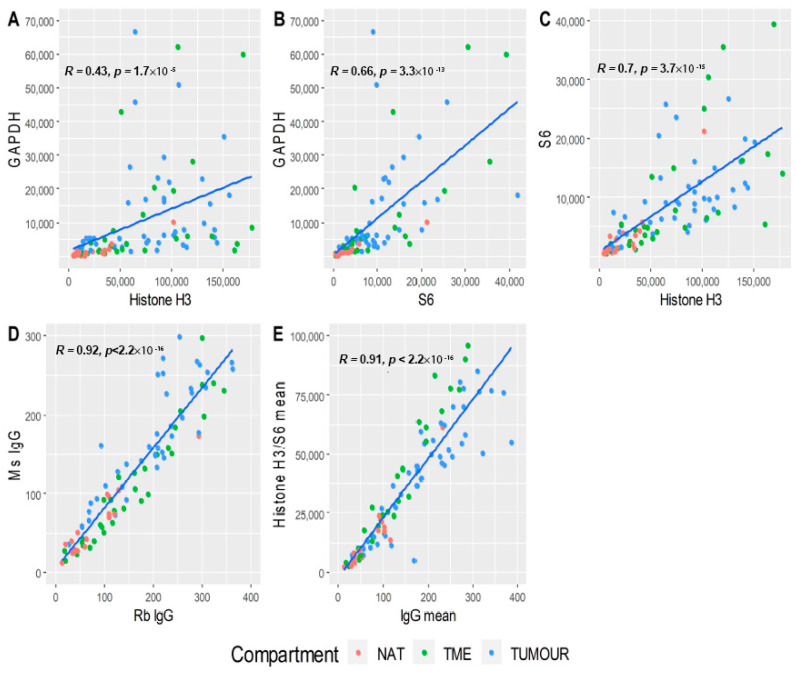
Assessment of housekeeping proteins and IgG as normalisers. Raw counts (**A**–**D**) and the means (**E**) of GAPDH, histone H3, S6, and Ms/Rb IgG were compared pairwise and linear regression performed to determine the Pearson correlation coefficient (*R*) between putative housekeepers. (**A**) GAPDH vs. histone H3, *R* = 0.43; (**B**) GAPDH vs. S6, *R* = 0.66; (**C**) histone H3 vs. S6, *R* = 0.7; (**D**) Ms IgG vs. Rb IgG, *R* = 0.92; (**E**) mean of Ms/Rb IgG vs. mean of histone H3/S6, *R* = 0.91. NAT: normal adjacent tissue; TME: tumour microenvironment; tumour: tumour region.

The method of normalisation between ROIs was assessed by examining correlations between histone H3, S6, GAPDH, and IgG background control probes, under the assumption that normalisers should correlate between ROIs and be unrelated to underlying biology. Housekeeping proteins included in the assay (GAPDH, histone H3, and S6) were plotted to determine which pairs best correlated across ROIs (Figure 4A–C). Histone H3 and S6 exhibited the strongest Pearson correlation coefficient (*R* = 0.7) (Figure 4C), and were thus examined further for correlation to IgG background to confirm independence from tissue biology. Ms and Rb IgG strongly correlated with each other across ROIs (*R* = 0.92) (Figure 4D), and the means of these IgG counts showed strong correlation with means of histone H3 and S6 housekeeping controls (*R* = 0.91), indicating that IgG controls, histone H3, and S6 were unrelated to underlying biology, and could act as appropriate normalisers across ROIs (Figure 4E).

**Figure 5:**
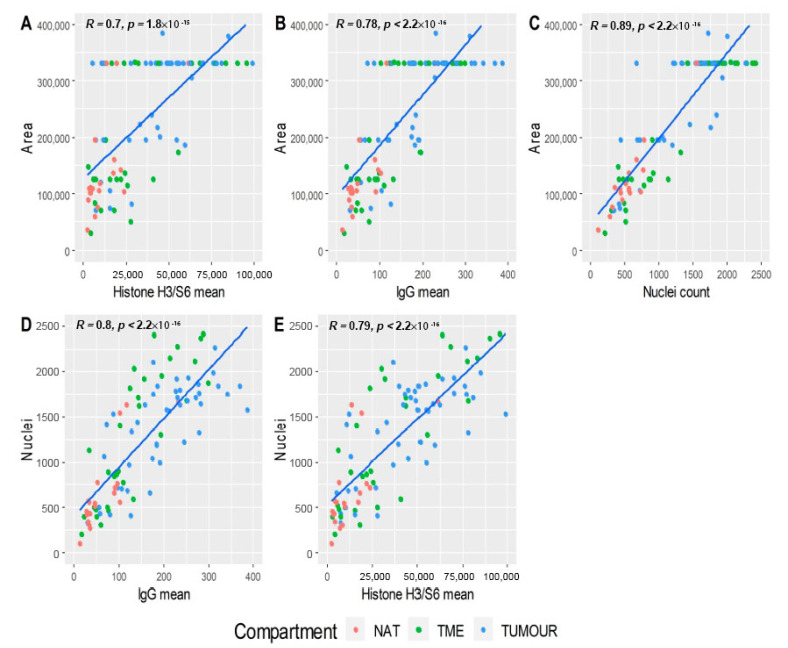
Assessment of ROI area and nuclei as normalisers. (**A**) ROI area was plotted against histone H3/S6 means (*R* = 0.7), (**B**) IgG means (*R* = 0.78), (**C**) and nuclei counts (*R* = 0.89). Nuclei counts were then evaluated against (**D**) IgG means (*R* = 0.8) and (**E**) histone H3/S6 means (R = 0.79). Some ROIs contained the maximum area (horizontal dots in A–C) and exhibited significant variance in the secondary parameter, indicating that area was not a suitable normalisation method. Nuclei counts (D–E) demonstrated a trend with the secondary parameter; however, correlation was not as significant as that observed for IgG or histone H3/S6. NAT: normal adjacent tissue; TME: tumour microenvironment; Tumour: Tumour region.

In addition to the normalisation by IgG and traditional housekeeping members, ROI area and nuclei count were inspected for their utility to normalise DSP data. Figure 5A–C illustrates the relationship that the ROI area possessed with histone H3/S6, IgG, and nuclei. A number of ROIs varied by area; however, some possessed maximum sized geometry, and these ROIs varied significantly in their relationship with other normalisation parameters, indicating that ROI area was not a useful normalisation method in this experiment. Similarly, nuclei counts were evaluated relative to IgG and histone H3/S6 means (Figure 5D,E), where some trend was evident but lacked the robustness of either IgG or histone H3/S6 normalisation. Histone H3/S6 means were therefore utilised for normalisation, and henceforth the comparative quantification of probes.

### 3.4. Data Analysis

**Figure 6:**
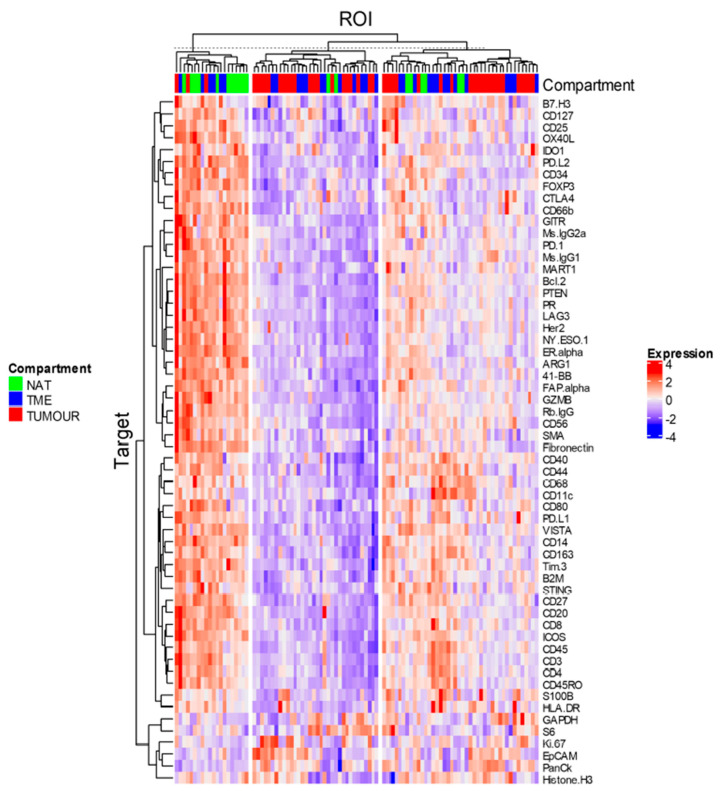
Clustered heatmap of relative expression of proteins per ROI. Ward D2 clustering was applied, followed by *K*-means clustering to delineate differences between expression profiles among compartments. NAT: normal adjacent tissue; TME: tumour microenvironment; tumour: tumour region.

Hierarchal clustering by the Ward D2 method [20] was first used to explore normalised data; however, expression appeared to vary significantly within classes of compartments, such that clear distinction between the NAT, tumour, and TME was not evident (Figure 6). *K*-means clustering to further group ROIs into classes showed most NATs grouping together (Figure 6, left), characterised by higher expression of most genes except for PanCK, EpCAM, and Ki-67. Another class consisting of both the TME and tumour (middle Figure 6) was characterised by lower expression of most proteins, with some ROIs expressing high levels of Ki-67 and EpCAM, whereas a third class was characterised by relatively heterogenous expression of all proteins (Figure 6, right).

Global correlation matrices for target protein expression within the TME (Figure S3) and tumour (Figure S4) indicated a large number of significant (*p* ≤ 0.001) positive correlations.

**Figure 7:**
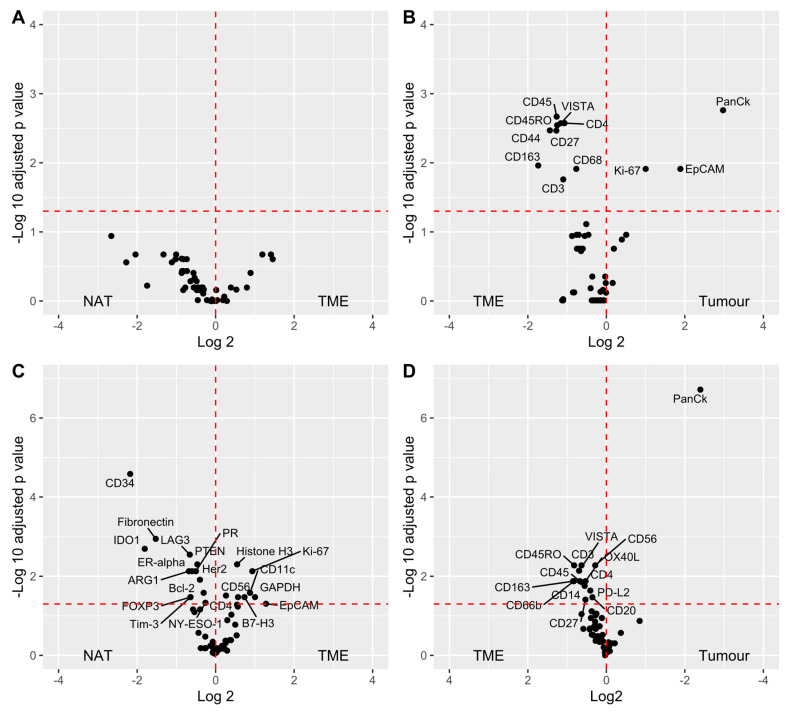
Differential expression of proteins between tissue compartments. Paired *t*-tests with a Benjamini–Hochberg correction were performed between matched compartments. Mann–Whitney tests with Benjamini–Hochberg correction were performed between unmatched compartments; *p-*values adjusted for multiple testing were used to identify significantly differentially expressed proteins. (**A**) NAT–TME (*n* = 8), (**B**) TME–tumour (*n* = 18), (**C**) NAT (*n* = 19) vs. TME (*n* = 32); (**D**) TME (*n* = 32) vs. tumour (*n* = 45). NAT: normal adjacent tissue; TME: tumour microenvironment; tumour: tumour region.

Differential protein expression was then evaluated between patient matched and unmatched compartments (Figure 7). Interestingly, matched TME and NAT (*n* = 8) did not exhibit significant differences (Figure 7A), while matched TME–tumour pairs (*n* = 18) indicated an expected enrichment of CD27, CD3, CD4, CD44, CD45, CD45RO, CD68, CD163, and VISTA within the TME, while tumour regions were enriched in Ki-67, EpCAM, and cytokeratin (Figure 7B).

**Figure 8:**
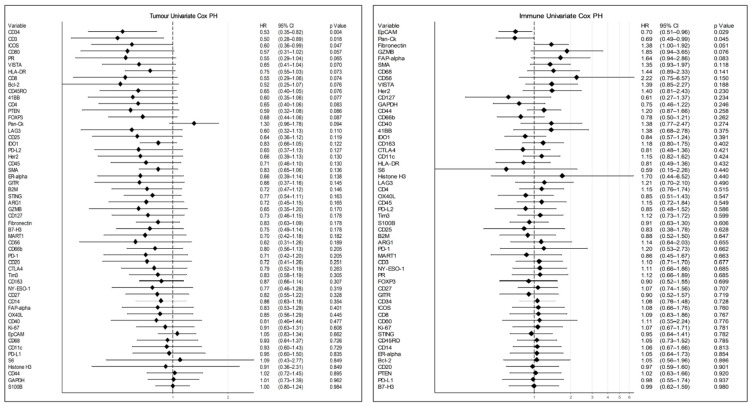
Cox proportional hazards of compartment-specific protein expression, ranked by association with overall survival. Log2 protein expression was modelled against follow-up time for the tumour and TME. Hazard ratio (HR) < 1 was associated with better patient outcome; HR > 1 was associated with poorer patient outcome.

When incorporating all samples, irrespective of patient pairing, several proteins appeared to be downregulated in TME relative to NAT, including CD34, fibronectin, IDO1, LAG3, ARG1, and PTEN (Figure 7C). TME–tumour comparisons remained similar to the paired data, whereas CD3, CD45RO, VISTA, and CD163 were enriched in the TME relative to the tumour (Figure 7D). Assessment of the association between protein expression and survival was also explored through an unadjusted, univariate Cox proportional hazards regression. Interestingly, expression data from immune ROIs indicated that the presence of EpCAM and cytokeratin was associated with better patient OS (Figure 8), while the presence of CD34, CD3, and ICOS in tumour ROIs was associated with better patient OS. When placed in a multivariate model to adjust for age, AJCC, and TNM tumour staging variables, those markers found to be significant in a univariate model no longer reached significance levels (data not shown). The number of samples did not permit higher-level multivariate analysis and statistical modelling of covariate prognostic signatures.

## 4. Discussion

The Nanostring GeoMx DSP platform [13,21] offers a novel solution for high-plex digital quantification of proteins and mRNA from fixed and fresh frozen tissues with spatial resolution [22]. It has been recently applied to triple-negative breast cancer (TNBC) [23], NSCLC [24], and melanoma [25]. However, the implementation and interpretation of such high-plex discovery is still in its infancy. The application of such technologies to large numbers of patient samples in the TMA format potentially provides unparalleled insight into spatial cell types, biomarkers, and the interactions that may underlie the disease biology. In this study, we quantified proteins across the current DSP immune cell profiling, IO drug targets, immune activation status, immune cell typing, and pan-tumour protein modules to understand the presence of these markers in tumours, tumour microenvironments, and histologically normal adjacent tissue compartments. We present a users’ experience where 96 ROIs were collected from a single TMA-containing tumour and NAT cores, with data processed and analysed within the GeoMx DSP analysis suite.

In conventional IHC and multiplex IHC, information can be obtained from the entirety of sections or TMA cores, giving a global perspective of marker expression and allowing post-hoc segmentation to inform on distribution. The DSP approach differs in that, while visualisation markers may inform on tumour/non-tumour regions and areas of immune cell infiltrate, ROIs are limited to a maximum of 600 µm geometric shapes. In this study, circular ROIs and several custom-drawn ROIs were used, meaning that “tumour” ROIs innately contained immune infiltrate, and that “immune” ROIs needed to be completely separate from the tumour to be defined, and may represent tumour-adjacent “stromal” immune infiltrate rather than an activated “tumour microenvironment” immune infiltrate. The DSP platform does allow for “masking” or “compartmentalization” within ROIs, enabling the signal to be obtained directly from tumour cells and from the immediate stromal space into which they have proliferated, at µm resolution [24]. However, this approach was not used in this study, and is a salient point to be considered for future analyses using the platform.

Here, we also demonstrate that there is a need to empirically determine the method of normalisation and identify probes which lack robust signal-to-noise. We demonstrated that both IgG background control probes as well as histone H3 and S6 housekeeping probes correlated across ROIs, while area and nuclei varied significantly and were thus less reliable for normalising data for quantification. Existing studies that have used area as a normaliser [24] have also utilised a signal-to-noise ratio cut-off >3, suggesting that our particular TMA may have exhibited disproportionately high background or low overall signal, as a significant number of probes were within range of IgG control probes. Without validation through IHC, it is difficult to interpret the meaning of probes that give signal within range of isotype IgG controls. It is noteworthy that, for example, PD-L1 counts fell below background where an abundance should be expected in a subset of NSCLC tissues, which highlights the importance of orthogonal validation when using a discovery technique. It is perhaps for this reason that many significant correlations were observed within compartments for markers that possessed low signal-to-noise, and these observations require additional validation.

It is important to note that the use of traditional methods of housekeeping normalisation in such datasets require deeper investigation. Evidence exists within our data for systematic lower expression in NAT samples, for which a single normalisation approach, including all samples will arbitrarily overestimate normalized NAT counts. This is critical in differential analysis where it should be assumed that most targets are not differentially expressed, and is better controlled for by global scaling methods, such as the “Trimmed Mean of M-values: (TMM) in edgeR package [26], and “Relative Log Expression” (RLE) in DESeq2 package [27]. Such methods require more advanced informatics processing beyond the DSP analysis suite.

With this in mind, it was notable that when differential analysis was applied by paired *t*-test to a limited number of patient pairs, NAT was indistinguishable from TME. A clear distinction between matched tumour and TME was evident, though, and was indicated by the increased presence of several key markers within the TME. Such markers included CD44, CD45, T cell lineage (CD3, CD4), memory T cells (CD45RO), monocyte/macrophage lineage (CD163, CD163), and costimulatory immune checkpoints (CD27, VISTA). When incorporating all samples, irrespective of patient matching, immunosuppressive molecules LAG3 [28] and IDO1 [29] were, perhaps counter-intuitively, significantly depleted in the TME relative to NAT, indicating the requirement for patient matching to make meaningful comparisons. Furthermore, mixed-model differential analysis should be performed to control for patient matching, where *t*-tests available for single-slide analysis within the DSP analysis suite are not wholly appropriate.

Nevertheless, the sheer scale of high-plex analyses appropriately applied to large numbers of cases through TMAs is an incredibly powerful tool for spatial biology. The DSP protein modules include key markers that describe multiple immune cell types, immune checkpoints, and experimental targets that enable a more comprehensive understanding of the immunological parameters that influence patient outcome. While overall survival was the only clinical endpoint investigated in this study, the emergence of patient cohorts treated with immunotherapies means that such assays may be used to track patient progression and outcomes, and indicate potential biomarkers for patients most likely to respond to these therapies. Despite some limitations in the absolute definition of tumour and TME compartments in our study, we were able to identify that the presence of CD3, CD34, and ICOS expressing cells in tumour compartments were associated with better patient OS in an unadjusted univariate Cox proportional hazards model. However, these findings require validation.

It is interesting to note that the enrichment of CD3 in tumour regions was associated with improved OS in this study, independently of CD4 T helper cells and cytotoxic CD8 T lymphocytes. Several markers significantly correlated with CD3 expression in the tumour compartment, including CD40, CD44, CD14, B2M, Tim-3, CD8, CD45RO, and ICOS, potentially implicating other cell lineages in immune-associated anti-tumour activity. Of note is the correlation between CD3 and ICOS, both of which were independently prognostic within tumour compartments, highlighting the potential power of such multiplex discovery.

Furthermore, limitations include the retrospective nature of the study, the need for orthogonal validation, and an increase in comparative groups.

## 5. Conclusions

In summary, the application of such novel platforms to provide comprehensive snapshots of clinical material enables an unprecedented insight into molecular phenotypes that may be indicative of response to emerging therapies, and ultimately, patient outcome. We propose the development of appropriate normalization methods to overcome systematic variation and low signal-to-noise, and indicate the requirement for larger sample numbers to overcome the limitations of multiple testing in discovery approaches. By combining such high-plex approaches with TMAs and orthogonal validation through multispectral IHC, a new field of biomarker discovery is developing that offers to change the way clinical pathology is performed.

## Figures and Tables

**Table 1:** Target proteins within Nanostring Digital Spatial Profiler (DSP) modules.

Controls	Immune Cell Profiling	IO Drug Target	Immune Activation Status	Immune Cell Typing	Pan-Tumour Module
Rb IgG	PD-1	4-1BB	CD127	CD45RO	MART1
Ms IgG1	CD68	LAG3	CD25	FOXP3	NY-ESO-1
Ms IgG2a	HLA-DR	OX40L	CD80	CD34	S100B
Histone H3	Ki-67	Tim-3	ICOS	CD66b	Bcl-2
S6	Beta-2M	VISTA	PD-L2	FAP-alpha	EpCAM
GAPDH	CD11c	ARG1	CD40	CD14	Her2
	CD20	B7-H3	CD44	CD163	PTEN
	CD3	IDO1	CD27		ER-alpha
	CD4	STING			PR
	CD45	GITR			
	CD56				
	CD8				
	CTLA4				
	GZMB				
	PD-L1				
	PanCk				
	SMA				
	Fibronectin				

**Table 2:** Patient characteristics of the TMA cohort by DSP tissue compartment.

Characteristic	Overall, *n* = 96	NAT, *n* = 19 ^1^	TME, *n* = 32 ^1^	Tumour, *n* = 45 ^1^	*p*-Value ^2^
Age	62 (54, 69)	66 (56, 69)	60 (54, 67)	62 (54, 71)	0.6
Sex					0.7
F	42 (44%)	9 (47%)	12 (38%)	21 (47%)	
M	54 (56%)	10 (53%)	20 (62%)	24 (53%)	
*t*					
0	19 (20%)	19 (100%)	0 (0%)	0 (0%)	
1	24 (25%)	0 (0%)	11 (34%)	13 (29%)	
2	34 (35%)	0 (0%)	15 (47%)	19 (42%)	
3	12 (12%)	0 (0%)	4 (12%)	8 (18%)	
4	7 (7.3%)	0 (0%)	2 (6.2%)	5 (11%)	
*n*					0.2
0	78 (81%)	19 (100%)	23 (72%)	36 (80%)	
1	13 (14%)	0 (0%)	6 (19%)	7 (16%)	
2	3 (3.1%)	0 (0%)	2 (6.2%)	1 (2.2%)	
3	2 (2.1%)	0 (0%)	1 (3.1%)	1 (2.2%)	

^1^ Statistics presented: median (IQR); *n* (%); ^2^ Statistical tests performed: Kruskal–Wallis test; chi-square test of independence; Fisher’s exact test.

## Supplementary Materials

The following are available online at https://www.mdpi.com/2072-6694/12/12/3551/s1, Figure S1: Range of probe counts from ROI-1 (Top) to ROI-96 (Bottom), Figure S2: Probe counts per compartment from most abundant (Top) to least abundant (Bottom), Figure S3: Pearson correlation matrix of proteins within TME ROIs and Figure S4: Pearson correlation matrix of proteins within tumour ROIs.

## References

[1] Siegel R.L., Miller K.D., Jemal A. (2020). Cancer statistics, 2020. CA Cancer J. Clin..

[2] Zappa C., Mousa S.A. (2016). Non-small cell lung cancer: Current treatment and future advances. Transl. Lung Cancer Res..

[3] Xia W., Yu X., Mao Q., Xia W., Wang A., Dong G., Chen B., Ma W., Xu L., Jiang F. (2017). Improvement of survival for non-small cell lung cancer over time. OncoTargets Ther..

[4] Kulasinghe A., Kapeleris J., Cooper C., Warkiani M.E., O’Byrne K.J., Punyadeera C. (2019). Phenotypic Characterization of Circulating Lung Cancer Cells for Clinically Actionable Targets. Cancers.

[5] Punyadeera C., Lim Y., Kapeleris J., Warkiani M.E., O’Byrne K.J., Punyadeera C. (2020). The Use of Three-Dimensional DNA Fluorescent in Situ Hybridization (3D DNA FISH) for the Detection of Anaplastic Lymphoma Kinase (ALK) in Non-Small Cell Lung Cancer (NSCLC) Circulating Tumor Cells. Cells.

[6] Samstein R.M., Lee C.-H., Shoushtari A.N., Hellmann M.D., Shen R., Janjigian Y.Y., Barron D.A., Zehir A., Jordan E.J., Omuro A. (2019). Tumor mutational load predicts survival after immunotherapy across multiple cancer types. Nat. Genet..

[7] Blanco-Melo D., Nilsson-Payant B.E., Liu W.-C., Uhl S., Hoagland D., Møller R., Jordan T.X., Oishi K., Panis M., Sachs D. (2020). Imbalanced Host Response to SARS-CoV-2 Drives Development of COVID-19. Cell.

[8] O’Callaghan D.S., Rexhepaj E., Gately K., Coate L., Delaney D., O’Donnell D.M., Kay E., O’Connell F., Gallagher W.M., O’Byrne K.J. (2015). Tumour islet Foxp3+ T-cell infiltration predicts poor outcome in nonsmall cell lung cancer. Eur. Respir. J..

[9] Swinson D.E., Jones J.L., Richardson D., Wykoff C., Turley H., Pastorek J., Taub N., Harris A.L., O’Byrne K.J. (2003). Carbonic anhydrase IX expression, a novel surrogate marker of tumor hypoxia, is associated with a poor prognosis in non-small-cell lung cancer. J. Clin. Oncol. Off. J. Am. Soc. Clin. Oncol..

[10] Guo X., Zhang Y., Zheng L., Zheng C., Song J., Zhang Q., Kang B., Liu Z., Jin L., Xing R. (2018). Global characterization of T cells in non-small-cell lung cancer by single-cell sequencing. Nat. Med..

[11] Welsh T.J., Green R.H., Richardson D., Waller D.A., O’Byrne K.J., Bradding P. (2005). Macrophage and Mast-Cell Invasion of Tumor Cell Islets Confers a Marked Survival Advantage in Non–Small-Cell Lung Cancer. J. Clin. Oncol..

[12] Giraldo N.A., Nguyen P., Engle E.L., Kaunitz G.J., Cottrell T.R., Berry S., Green B., Soni A., Cuda J.D., Stein J.E. (2018). Multidimensional, quantitative assessment of PD-1/PD-L1 expression in patients with Merkel cell carcinoma and association with response to pembrolizumab. J. Immunother. Cancer.

[13] Beechem J. (2019). High-Plex Spatially Resolved RNA and Protein Detection Using Digital Spatial Profiling: A Technology Designed for Immuno-oncology Biomarker Discovery and Translational Research. Methods in Molecular Biology.

[14] RStudio Team (2020). RStudio: Integrated Development for R.

[15] Daniel D., Sjoberg M.H., Whiting K., Zabor E.C. (2020). Gtsummary: Presentation-Ready Data Summary and Analytic Result Tables. http://www.danieldsjoberg.com/gtsummary/.

[16] Wickham H. (2016). ggplot2: Elegant Graphics for Data Analysis.

[17] Kassambara A. (2020). ggpubr: ‘ggplot2′ Based Publication Ready Plots. https://rpkgs.datanovia.com/ggpubr/.

[18] Gu Z., Eils R., Schlesner M. (2016). Complex heatmaps reveal patterns and correlations in multidimensional genomic data. Bioinformatics.

[19] Wiesweg M. (2020). SurvivalAnalysis: High-Level Interface for Survival Analysis and Associated Plots. https://rdrr.io/cran/survivalAnalysis/.

[20] Murtagh F., Legendre P. (2011). Ward’s hierarchical clustering method: Clustering criterion and agglomerative algorithm. arXiv.

[21] Merritt C.R., Ong G.T., Church S.E., Barker K., Danaher P., Geiss G., Hoang M., Jung J., Liang Y., McKay-Fleisch J. (2020). Multiplex digital spatial profiling of proteins and RNA in fixed tissue. Nat Biotechnol..

[22] Nerurkar S.N., Goh D., Cheung C.C.L., Nga P.Q., Lim J.C., Yeong J.P. (2020). Transcriptional Spatial Profiling of Cancer Tissues in the Era of Immunotherapy: The Potential and Promise. Cancers.

[23] Stewart R.L., Matynia A.P., Factor R.E., Varley K.E. (2020). Spatially-resolved quantification of proteins in triple negative breast cancers reveals differences in the immune microenvironment associated with prognosis. Sci. Rep..

[24] Zugazagoitia J., Gupta S., Liu Y., Fuhrman K., Gettinger S., Herbst R.S., Schalper K.A., Rimm D.L. (2020). Biomarkers Associated with Beneficial PD-1 Checkpoint Blockade in Non-Small Cell Lung Cancer (NSCLC) Identified Using High-Plex Digital Spatial Profiling. Clin. Cancer Res..

[25] Toki M.I., Merritt C.R., Wong P.F., Smithy J.W., Kluger H.M., Syrigos K.N., Ong G.T., Warren S.E., Beechem J.M., Rimm D.L. (2019). High-Plex Predictive Marker Discovery for Melanoma Immunotherapy–Treated Patients Using Digital Spatial Profiling. Clin. Cancer Res..

[26] Robinson M.D., McCarthy D.J., Smyth G.K. (2009). edgeR: A Bioconductor package for differential expression analysis of digital gene expression data. Bioinformatics.

[27] Anders S., Huber W. (2010). Differential expression analysis for sequence count data. Genome Biol..

[28] Xu F., Liu J., Liu D., Liu B., Wang M., Hu Z., Du X., Tang L., He F. (2014). LSECtin expressed on melanoma cells promotes tumor progression by inhibiting antitumor T-cell responses. Cancer Res..

[29] Spranger S., Koblish H.K., Horton B., Scherle P.A., Newton R., Gajewski T.F. (2014). Mechanism of tumor rejection with doublets of CTLA-4, PD-1/PD-L1, or IDO blockade involves restored IL-2 production and proliferation of CD8(+) T cells directly within the tumor microenvironment. J. Immunother. Cancer.

