# Supplementary material for: High-Plex and High-Throughput Digital Spatial Profiling of Non-Small-Cell Lung Cancer (NSCLC)
Source: Cancers (Basel). 2020 Nov 27;12(12):3551. doi: 10.3390/cancers12123551 (PMC7760230; doi:10.3390/cancers12123551)
Supplement: Supplementary file 1 [file cancers-12-03551-s001.pdf]

Supplementary Materials:

High-Plex and High-throughput Digital Spatial Profiling of Non-Small-Cell Lung Cancer (NSCLC)

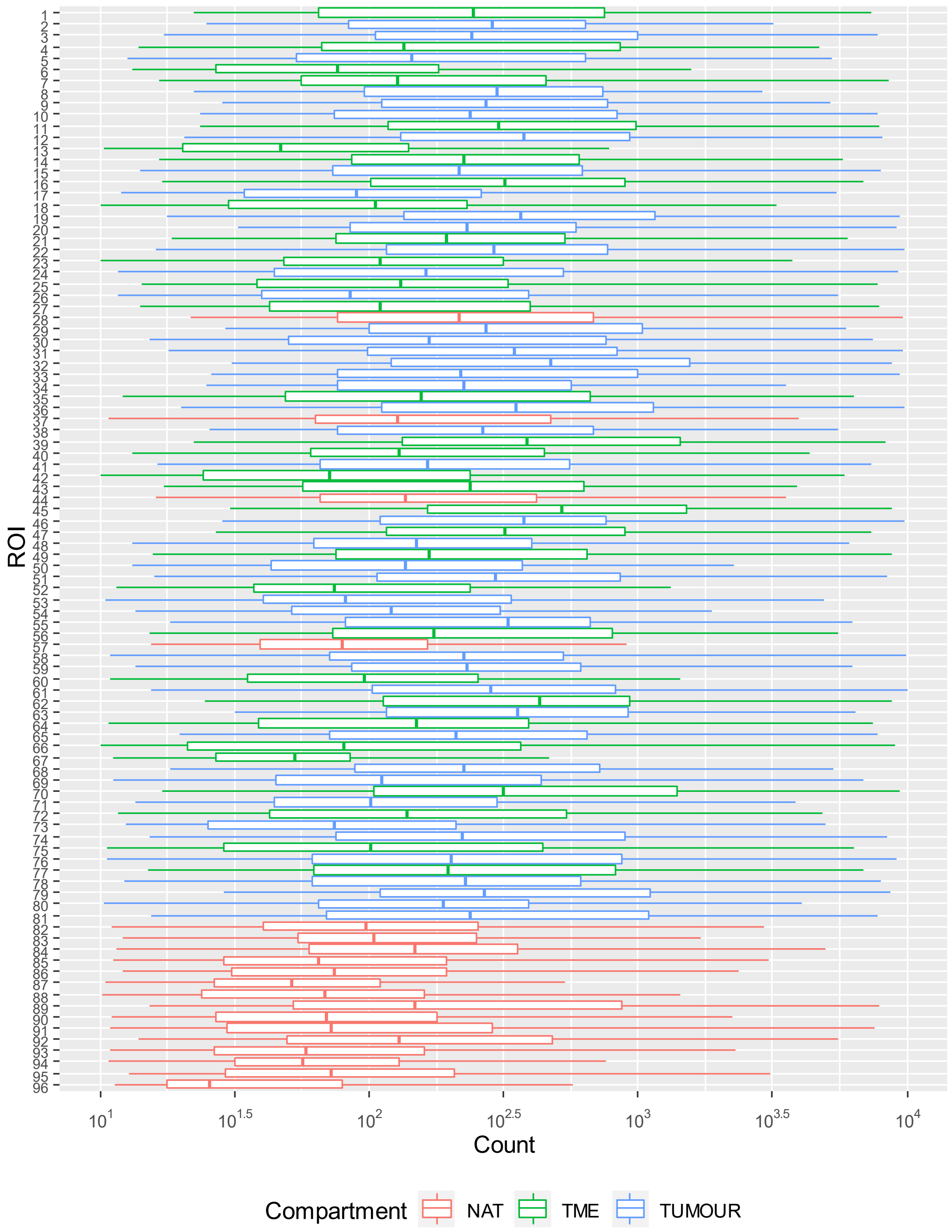

Figure S1. Range of probe counts from ROI-1 (Top) to ROI-96 (Bottom). Raw counts for all probes per ROI were plotted to confirm comparable ranges of signal between ROIs. NAT: Normal adjacent tissue; TME: Tumour microenvironment; Tumour: Tumour region.

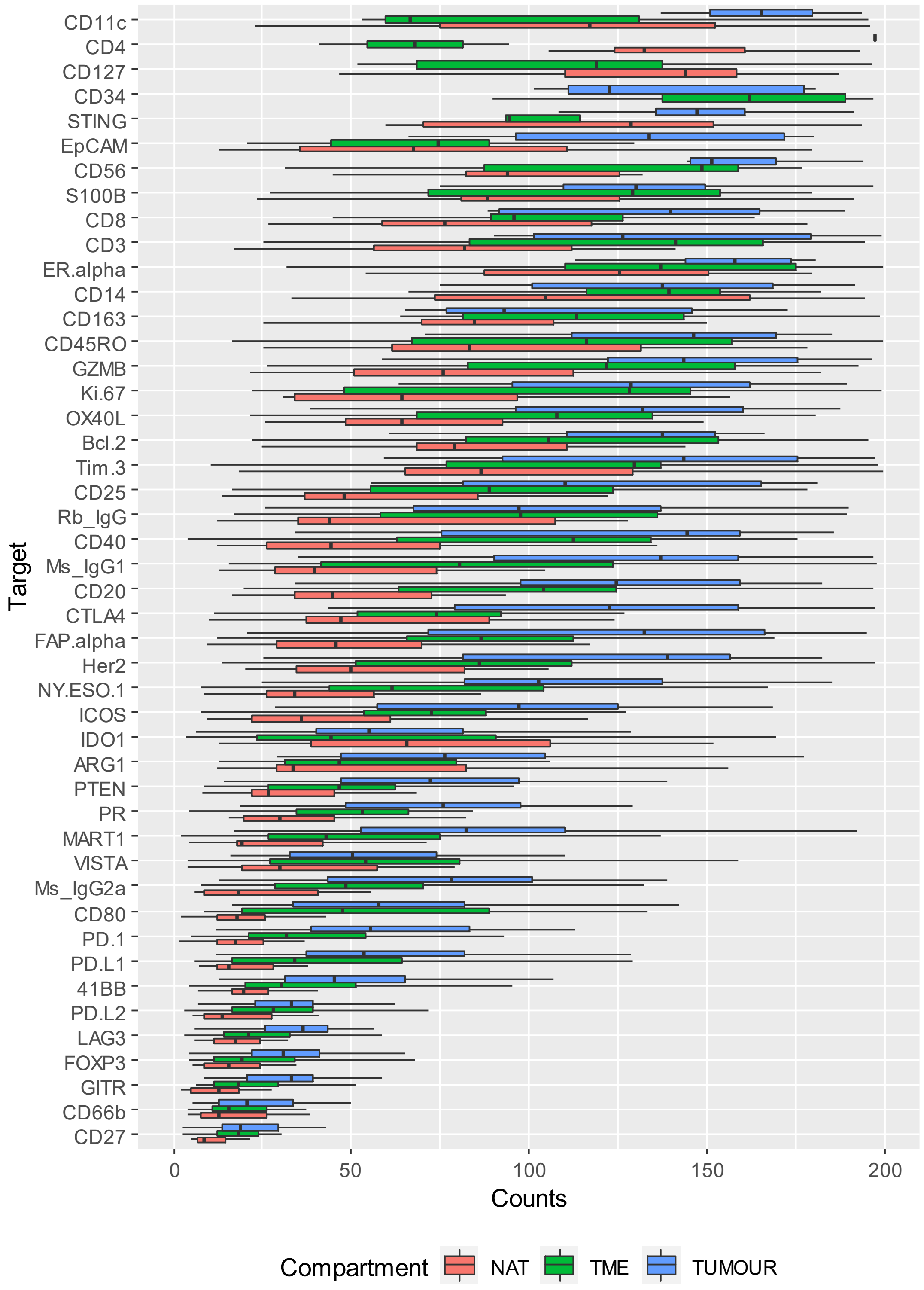

Figure S2. Probe counts per compartment from most abundant (Top) to least abundant (Bottom). Raw counts for each probe were plotted to examine compartment specific expression and quantifiable range of lowly expressed targets. Counts for highly abundant Histone H3, SMA, S6, GAPDH, fibronectin, cytokeratin, CD44, CD68, β-2-microglobulin (B2M), HLA-DR, CD45 and B7-H3 excluded here. NAT: Normal adjacent tissue; TME: Tumour microenvironment; Tumour: Tumour region.

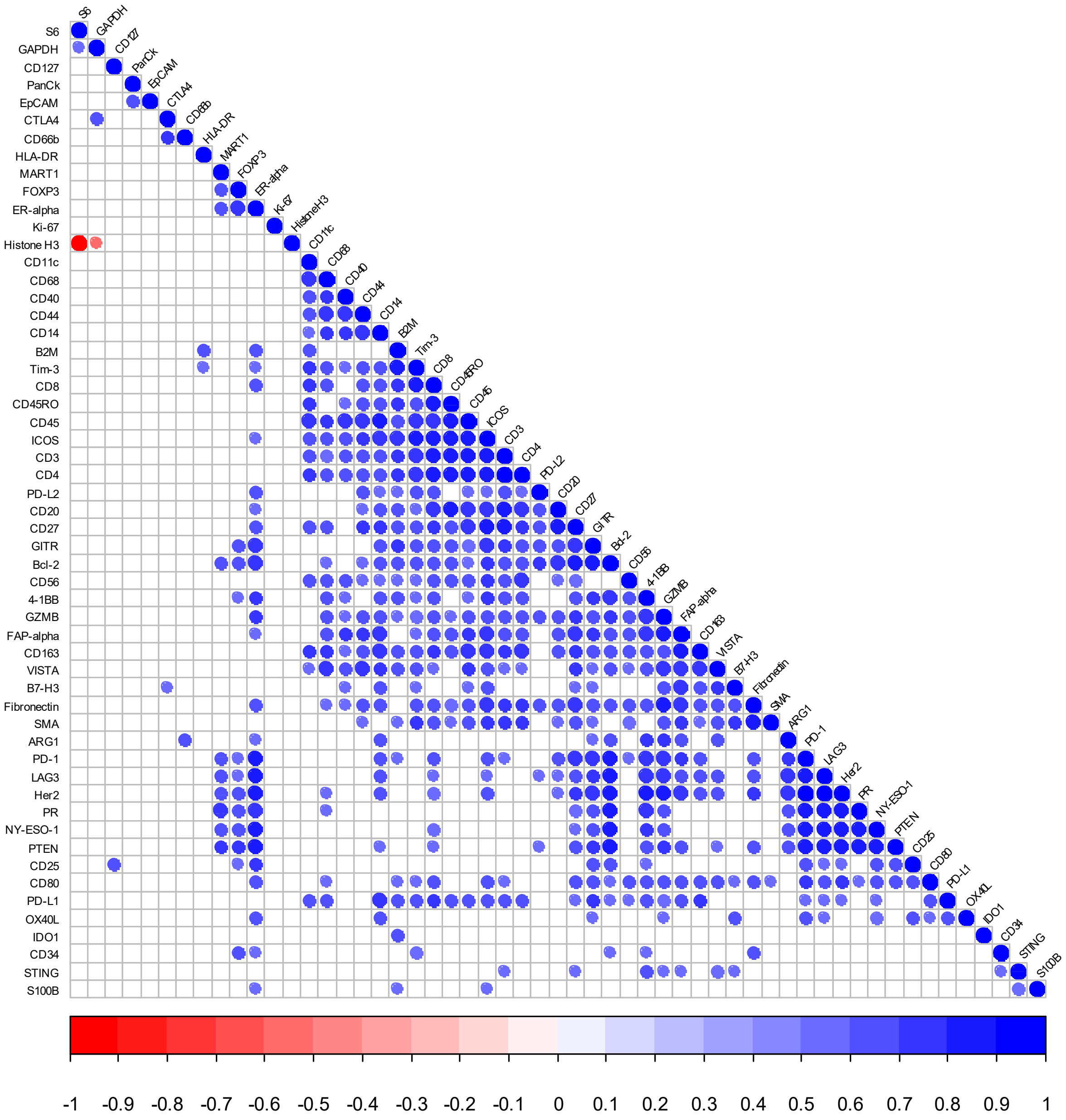

Figure S3. Pearson correlation matrix of proteins within TME ROIs. Log_2 protein expression was used to evaluate Pearson correlations between proteins. Only correlations with p value <0.001 are shown. Blue= positive correlation, Red= negative correlation

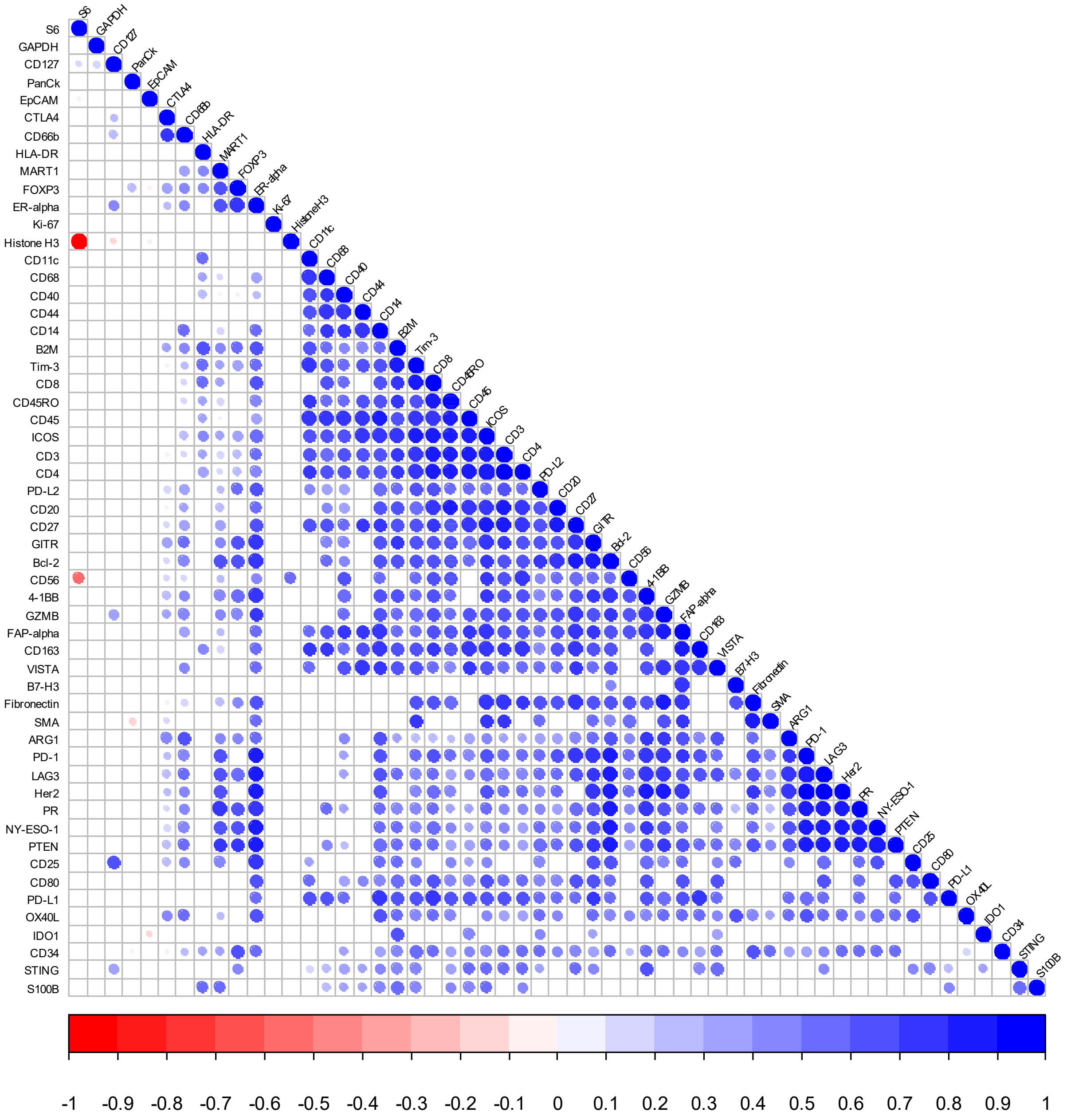

Figure S4. Pearson correlation matrix of proteins within tumour ROIs. Log_2 protein expression was used to evaluate Pearson correlations between proteins. Only correlations with *p* value <0.001 are shown. Blue= positive correlation, Red= negative correlation.
